# CARF regulates the alternative splicing and piwi/piRNA complexes during mouse spermatogenesis through PABPC1: CARF regulates spermatogenesis through PABPC1

**DOI:** 10.3724/abbs.2024224

**Published:** 2024-12-11

**Authors:** Yuming Cao, Shengnan Wang, Jie Liu, Jinfeng Xu, Yan Liang, Fei Ao, Zexiao Wei, Li Wang

**Affiliations:** Department of Obstetrics and Gynecology Perinatal Medical Center the Fifth Affiliated Hospital of Sun Yat-sen University Zhuhai 519000 China

**Keywords:** alternative splicing, CARF, spermatogenesis, PABPC1, male infertility

## Abstract

ADP-ribosylation factor collaborator (CARF), which is also known as CDKN2AIP, was first recognized as an ADP-ribosylation factor-interacting protein that participates in the activation of the ARF-p53-p21 (WAF1) signaling pathway under different conditions, such as oxidative and oncogenic stresses. The activation of this pathway often leads to cell growth arrest and apoptosis as well as senescence. Previous studies revealed that CARF, an RNA-binding protein, is critical for maintaining stem cell pluripotency and somatic differentiation. Nevertheless, its involvement in spermatogenesis has not been well examined. In this study, we show that male mice deficient in *Carf* expression present impaired spermatogenesis and fertility. IP-MS and RNA-seq analyses reveal that CARF/ *Carf* interacts with multiple key splicing factors, such as PABPC1, and directly targets 356 different types of mRNAs in spermatocytes. *Carf*-associated mRNAs display aberrant splicing patterns when Carf expression is deficient. In addition, our results demonstrate that PIWIL1 expression and localization are altered in the *Carf* ^*-*/ *-*^ mouse model through the downregulation of PABPC1, which further affects the ratio of pachytene-piRNA. Our study suggests that CARF is critical for regulating alternative splicing in mammalian spermatogenesis and determining infertility in male mice.

## Introduction

Spermatogenesis refers to the dynamic biological development that takes place in seminiferous tubules [1]. Spermatogonia can self-renew and de-different into spermatogonial stem cell to replenish the pool of stem cells [2]. Type B spermatogonia are generated from type A spermatogonia before they develop into spermatocytes through mitosis and proliferation. Round spermatids, which are the first haploid cells, are subsequently generated after primary spermatocyte division. Finally, round spermatids undergo sperm deformation to form spermatozoa [ 3, 4]. This complicated physiological process is precisely maintained and regulated by several genes at different levels [ 5– 8]. Alternative splicing has been well demonstrated to occur particularly in the testis [9]. However, how this process affects spermatogenesis is not well understood. RNA-binding proteins can not only regulate alternative splicing of mRNAs but also participate in their transportation and translation and play a key role in spermatogenesis [10]. In fact, accumulating evidence suggests that RNA-binding proteins may play a more important role in regulating exon inclusion and protein expression than previously thought [11].

The functions of many RNA-binding proteins have been well studied; for example, poly-A binding protein cytoplasmic 1 (PABPC1) is abundantly expressed in round spermatids before elongation, during which it regulates the expression of an alternative exome that is indispensable for the completion of sperm maturation [ 12, 13]. Spermatogenetic downregulation of RNA-binding proteins, such as PABPC1, is known to be associated with greater befit from retroelements, especially during the elongation of spermatids [14]. Exploring the upstream and downstream proteins of PABPC1 is also the main way to analyze the process of spermatogenesis [15].

Alternatively, in the haploid phase of spermatogenesis, p-element-induced wimpy testis like 1 (PIWIL1) is known to be associated with maintaining normal sperm development in the translational regulation of post-meiotic mRNAs [16]. PIWIL1 binds to PABPC1 and collectively regulates spermatogenesis [17]. If the expression and function of any of these proteins are affected, it can lead to development abnormalities and cause spermatogenesis disorders [ 18, 19].

Many epigenetic mechanisms, including alternative splicing, which is among the most important, have been proposed to regulate transcription activity. After alternative splicing, a single gene can be transcribed into a variety of mRNA and protein isoforms that present different or even contradictory functional and structural characteristics [20]. A previous study indicated that during spermatogenesis, alternative pre-mRNA splicing is critical for the transcriptional regulation of gene expression and function [21]. Recent high-throughput analyses have shown that alternative splicing can occur in more than 95% of all human genes and 60% of mouse genes [22]. Several patterns of alternative splicing events have been identified, such as exon skipping (ES), intron retention (IR), alternative first exon (AFE) as well as mutually exclusive exon (MXE) [23]. Previous studies have shown that aberrant alternative splicing of genes related to reproductive development could result in impaired spermatogenesis and male infertility.

When it was first discovered, CARF was considered an alternative reading frame-interacting protein [24] and could inhibit the transcription of *HDM2* as well as P53 signaling pathways [25]. Numerous studies have demonstrated the importance of CARF in spermatogenesis. For example, studies have shown that the number and quality of spermatozoa at 8 weeks of age are significantly reduced in *Carf* ^*–*/ *–*^ mice, mainly due to the abnormal process of protamine replacement of histones [26]. Similarly, in aged mice, Wnt signaling in Sertoli cells and undifferentiated spermatogonia was significantly decreased in 18-month-old knockout mice, which resulted in germ cell loss and decreased fertility [27]. Unfortunately, the transcriptional regulatory mechanism of *Carf* during spermatogenesis has not been fully elucidated. In the present study, we aimed to explore the underlying mechanism of alternative splicing in regulating spermatogenesis as well as male fertility in *Carf* ^*–* / *–*^ mice.

## Materials and Methods

### Database evaluation

We applied the Multiple Sequence Alignment online website ( https://www.ebi.ac.uk/Tools/msa/) to conduct homology analysis of CARF among various species. SMART online software ( https://smart.embl-heidelberg.de/) was used to analyze the CARF domain. CARF function analysis was performed via Hitpredict online software and ZhangLab ( http://www.hitpredict.org; https://zhanggroup.org/). The expression profiles of *Carf* in germ cells from different cell types at different developmental stages were analyzed via the Male Health Atlas single-cell sequencing database (MHA: Male Health Atlas).

### Experimental model

The protocols for experiments involving the mice used in this study were approved by the ethics Committee of Wuhan University (Approval No. WP2020-08005) and were carried out following instructions from the Institutional Animal Care and Use Committee of Wuhan University. The mice were kept in a SPF room with a constant temperature of 20°C to 25°C and 12/12 light/dark cycles as well as constant 55% humidity and free access to food and water.

*Carf*-knockout mice with a C57BL/6J genetic background were generated via the CRISPR‒Cas9 technique by Cyagen Biosciences (Suzhou, China). The DNA fragments from exons 1 to 3 were deleted. The knockout mice were genotyped for *Carf* expression via polymerase chain reaction (PCR) and DNA sequencing analysis. The sequences of the primers used in this study are presented in Supplementary Table S1.

### H&E staining

Unilateral testes and epididymides from wild-type and *Carf* ^*–*/ *–*^ mice were collected immediately after euthanization and fixed with a solution containing 4% paraformaldehyde (P1110; Solarbio, Beijing, China) at 4°C for 12 h. The experiments were repeated at least three times. Afterwards, the tissues were dehydrated in 70% ethanol and embedded in paraffin wax. Sections (5-μm-thick) were cut from the blocks at 50 μm intervals. After deparaffinization, the slides were stained with hematoxylin and eosin (H&E) solution (G1120-3; Solarbio) following the manufacturer’s protocol. Histological images were captured via an integrated fluorescence microscopy imaging system (BZ-X800E; KeyGEN, Nanjing, China).

### Immunofluorescence analysis

Paraffin sections of testicular tissue were prepared via the same method used for HE staining. After being washed in PBS in 0.5% Triton X-100 three times, the slides were boiled in citrate antigen retrieval solution for 30 min and then blocked for 1 h with PBS containing 10% goat serum at room temperature. The primary antibodies listed in Supplementary Table S2 were applied, and the slides were incubated overnight at 4°C. The slides were then rinsed with PBS 3 times and stained with the appropriate fluorochrome-conjugated secondary antibodies (1:400, ab150077; Abcam, Cambridge, UK) at room temperature for 1 h. After rinsing, the nuclei were visualized via DAPI staining (ZLI-9557; ZSGB-BIO, Beijing, China). A fluorescence microscope (BX53; OLYMPUS, Tokyo, Japan) was used for the observation of the slides.

### RNA extraction and quantitative RT-PCR (qPCR)

The total RNA of the mouse testes was extracted with an RNA extraction kit (Cat#: RK30120; ABclonal, Wuhan, China) following the manufacturer’s protocol. RNase R (3 U/mg; Epicenter, Madison, USA) was applied to the sample for 15 min at 37°C as indicated, and qPCR was then applied to assess expression stability. Next, the mRNAs were converted into cDNA via an RT Master Mix Kit (Cat# R323-01; Vazyme, Nanjing, China) for qPCR following the manufacturer’s protocol. Next, target gene expression was determined via SYBR® Green qPCR Master Mix (Cat#: Q221-01; ABclonal). *GAPDH* was used as the housekeeping gene. The 2 ^–ΔΔCt^ method was applied to determine the relative gene expression. Supplementary Table S1 lists the sequences of the primers used in this study.

### Western blot analysis

Proteins were extracted from 20-week-old mouse testicular tissue. Briefly, 20 μg of protein sample was loaded onto a gel for electrophoresis. After being transferred to polyvinylidene fluoride membranes, the membranes were incubated with primary antibodies overnight in a cold room. After rinsing, the membrane was stained with horseradish peroxidase (HRP)-conjugated secondary antibodies at room temperature for one hour and visualized with an enhanced chemiluminescent substrate (Cat# RM00021; ABclonal) after rinsing. The data were analyzed via ImageJ software (1.8.0_112; National Institutes of Health, Bethesda, USA). The expression levels of the target proteins were normalized to that of GAPDH. The antibodies used in the experiment are described in Supplementary Table S2.

### Immunohistochemical (IHC) staining

Slides were deparaffinized in xylene and rehydrated in ethanol, and the antigen was retrieved with citric acid antigen repair solution (pH = 6, Cat No. #C9999; Sigma-Aldrich, St Louis, USA). Anti-rabbit PABPC1 (1:100, Cat No. #53348; Cell Signaling, Beverly, USA) and CARF (1:100, Cat No. #16615-1-AP; Proteintech) primary antibodies were used for staining overnight. Next, the samples were incubated with 3,3′-diaminobenzidine (Macklin, Shanghai, China) and counterstained with hematoxylin. An Olympus microscope (BX53; OLYMPUS) was used for imaging, and ImageJ software was used for quantification.

### RNA-seq analysis

Unilateral testes from three 20-week-old wild-type and *Carf* ^*–*/ *–*^ mice were obtained and stored in liquid nitrogen. TRIzol reagent (Invitrogen, Carlsbad, USA) was used to extract the RNA [26]. The quantification and quality control of the total RNA were conducted via a NanoDrop and an Agilent 2100 Bioanalyzer (Thermo Fisher Scientific, Waltham, USA). The library was construct by Novogene (Tianjin, China), which was subsequently sequenced.

### Immunoprecipitation (IP) analysis

Protein extracts of the testis were prepared in IP buffer (P0013J; Beyotime, China) supplemented with a protease inhibitor cocktail (CW2200S; CWBIO, Taizhou, China) for 30 min at 4°C and cleared by centrifugation. Protein lysates were incubated with antibodies or control IgG with rotation at 4°C overnight. Then, 25 μL of protein A Dynabeads (10001D; Thermo Fisher Scientific) and a sufficient amount of protein G Dynabeads (10003D; Thermo Fisher Scientific) were added to the mixture and incubated for an additional 6 h. The immunoprecipitates were separated by SDS-PAGE, and ruby fuel was applied for dyeing according to the product manual (S12000; Thermo Fisher Scientific). Next, the significantly different bands from the two groups were cut for protein mass spectrometry identification by mass spectrum.

Co-immunoprecipitation of CARF with PABPC1 was performed via SDS-PAGE, and the proteins were subjected to western blot analysis.

### Bioinformatics analysis

Total RNA was extracted from P16 (more pachytene spermatocytes to obtain a sufficient number of mature piRNAs) wild-type and *Carf* ^*–*/ *–*^ testes via TRIzol (Cat No. #15596026CN; Thermo Fisher Scientific). A total of 15–50 nucleotides (nt) were selected from the total RNA and transferred to a 15% TBE-urea gel. The concentration and quality of each sample were assessed via NanoDrop and RT-PCR. The results mapping to the genome (mm10) were included for additional analysis. Next, the small RNA sequencing data were deposited in the NCBI database under the accession number PRJNA398115.

### Statistical analysis

Individuals who conducted the analyses were blinded to the study design. GraphPad Prism software (V9; GraphPad Software, San Diego, USA) was used for the statistical analyses. The data were first tested for normality and examined via one-way or two-way analysis of variance. Tukey’s post hoc test was then applied to all analyses. Statistical significance was defined as a *P* value less than 0.05.

## Results

### CARF is highly conserved and critical in mRNA splicing

We performed conservation analyses using CARF protein sequences from different species, and the results revealed that the CARF protein sequence is highly conserved. It is highly homologous between humans and mice, with an 86.94% conserved sequence ( Supplementary Figure S1A). The CARF protein contains two strong RNA binding domains, XTBD and DSRM ( Supplementary Figure S1B). CARF plays a crucial role in both humans and mice. The 3D structure database has predicted the structure of CARF amino acids 1--580 in humans and CARF amino acids 1-563 in mice ( Supplementary Figure S1C,D). The human protein atlas database (HitPredit) indicates that the function of CARF is related mainly to transcriptional regulation of mRNA splicing ( Supplementary Figure S1E) and that it is involved mainly in the regulation of the cell cycle process ( http://www.hitpredict.org; Supplementary Figure S1F). These results suggest that CARF may regulate transcription by regulating the variable splicing of key gene mRNAs.

### CARF is expressed mainly in spermatocytes and spermatids

**Figure 1:**
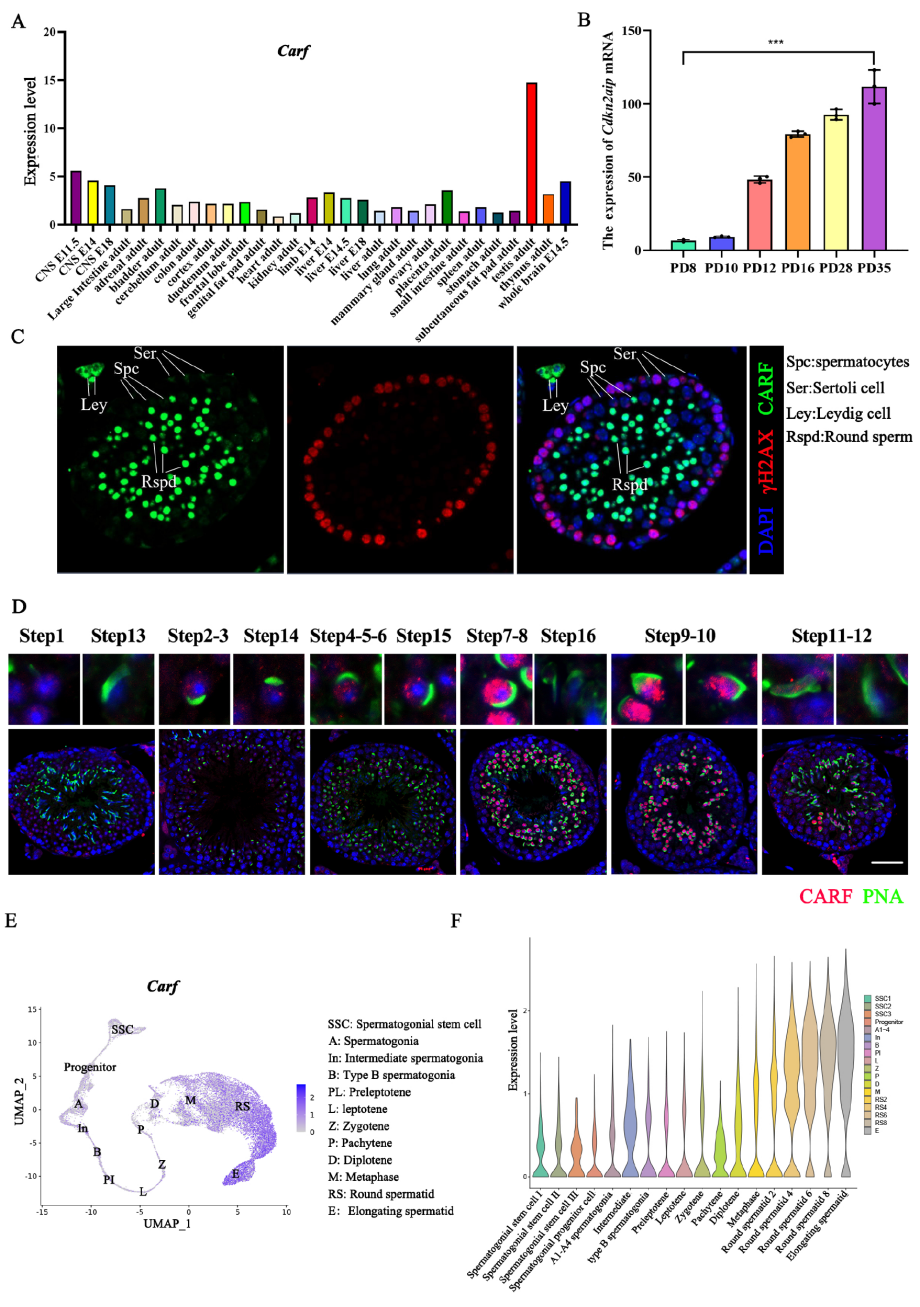
Figure 1 CARF is highly expressed in spermatocytes and spermatids of the testis (A) NCBI database analysis of the expression profile of Carf mRNA in mouse tissues. (B) qPCR analyses of Carf mRNA levels in multiple organs of mice. Data are presented as the mean ± SEM, n = 3. (C) Immunofluorescence staining analysis of CARF (green) in testis sections. γH2AX (red) was used as a marker for spermatocytes. The nuclei were stained with DAPI (blue). Spc indicates spermatocytes, Ser indicates Sertoli cells, Ley indicates Leydig cells, and Rspd indicates round sperm. Scale bar: 50 μm. (D) Immunofluorescence staining analysis of CARF (red) and PNA (green) in testis sections. The nuclei were stained with DAPI (blue), Scale bar: 50 μm. (E,F) The expression pattern of CARF in the mouse germline atlas was analyzed via a single-cell sequencing database (http://malehealthatlas.cn/ ).

According to the NCBI database, the *Carf* gene is highly expressed in mouse testicular tissue ( Figure 1A). Furthermore, the qPCR results revealed that *Carf* mRNA preferentially expressed in the testis rather than in other organs ( Figure 1B), following a similar pattern as we previously discovered with the CARF protein. To further explore the localization of CARF, frozen mouse testis sections were prepared and co-stained with γH2AX (a marker for DSBs) and CARF. We also co-stained two primary antibodies from different companies with PNA to further verify the localization of CARF in mouse testicular germ cells ( Figure 1C). The results indicated that most CARF expression was localized in round spermatozoa and elongated spermatocytes, whereas weak expression was observed in Sertoli cells and primary spermatocytes ( Figure 1D). This result was consistent with the single-cell database ( http://malehealthatlas.cn/) of the mouse testis development atlas, which revealed that CARF was expressed mainly in round spermatozoa at different stages of development ( Figure 1E,F and Supplementary Figure S2A,B). This specific expression pattern of CARF shows that it may play critical roles in spermatogenesis.

### *Carf* is essential for spermatogenesis

**Figure 2:**
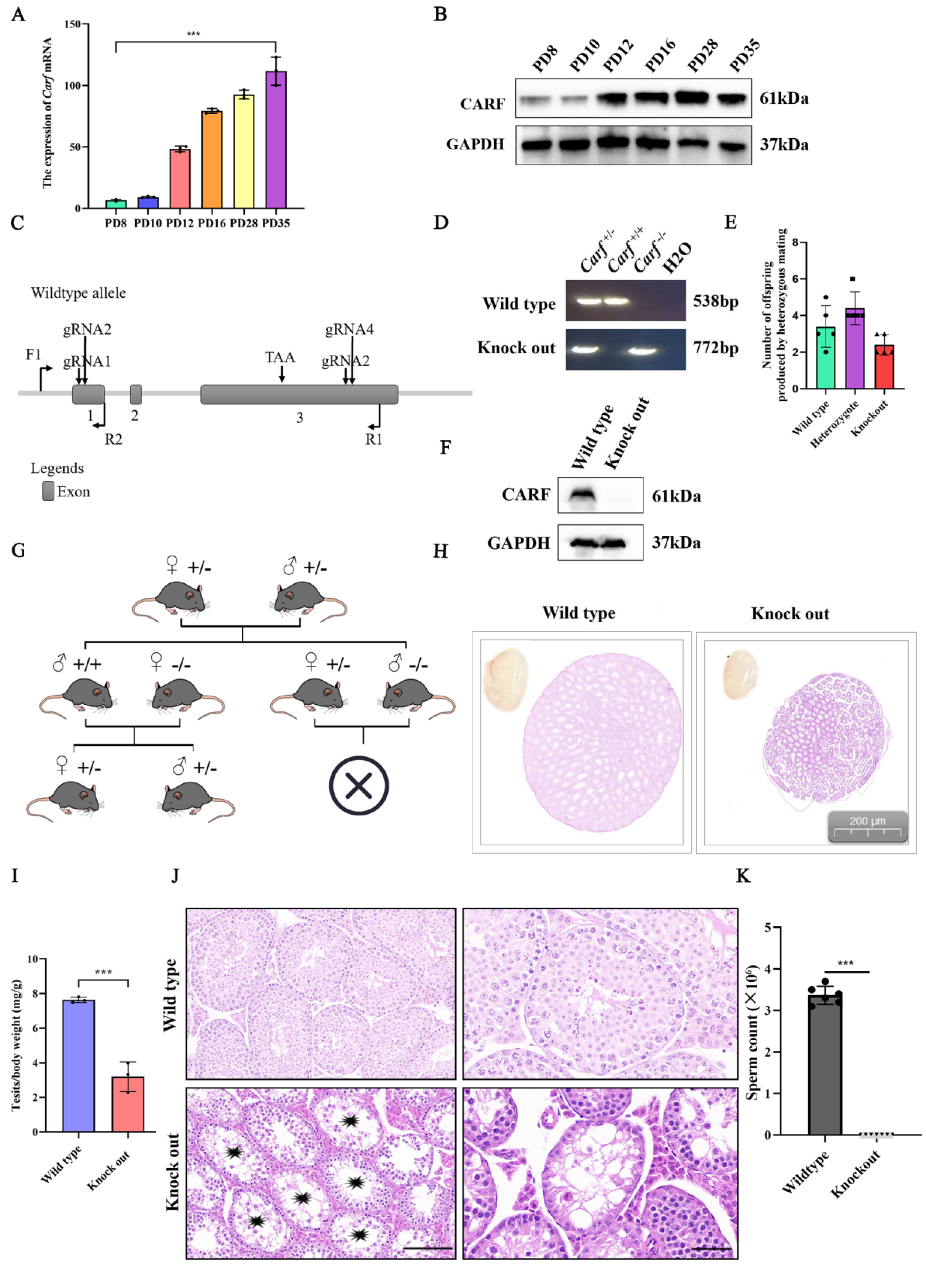
Figure 2 CARF is essential for germ cell development (A) Expression of Carf mRNAs in developing testes at postnatal day 8 (P8), P10, P12, P16, P28 and P35 were analyzed via qPCR. Data are presented as the mean ± SEM, n = 3. (B) Expression of the CARF protein in developing testes at postnatal day 8 (P8), P10, P12, P16, P28 and P35 were analyzed via western blot analysis. (C) Schematic of the generation of Carf–/–-deficient mice with the CRISPR-Cas9 genome editing system. Four small guide RNAs (sgRNAs) were designed to generate Carf-knockout mice. The DNA fragments covering exon 1 to exon 3 were deleted. (D) Representative image of PCR genotyping via the F1, R1 and R2 primers. Heterozygotes (+/–), wild-type (+/+) and knockout (–/–) alleles generate PCR products of 538 bp and 772 bp, 538 bp, and 772 bp, respectively. H2O was used as a negative control for the PCR. M, 1000 bp marker. (E) Litter size produced by heterozygotes (n = 5/group). Data are presented as the mean ± SEM. (F) Western blot analysis of CARF protein expression in testis extracts from wild-type and Carf-knockout testes at P56. GAPDH served as a loading control. (G) The mating strategy of heterozygous mice was studied to analyze the litter size of female and male mice after mating. (H) Gross morphology of testes from wild-type and Carf –/– mice at the age of 20 weeks. Panoramic images of testicular tissue were scanned with ImageScope software. (I) Testis weight/body weight ratio of 20-week-old wild-type and Carf-knockout male mice. The Carf-knockout male mice presented significantly reduced testis weight (42% of wild-type) (P < 0.001, n = 6). (J) Testicular histology of wild-type and Carf–/– mice at the age of 20 weeks. The number of germ cells in Carf –/– male mice was significantly lower than that in wild-type male mice. The scale bar of the picture in the left panel is 100 μm, whereas the scale bar of the picture in the right panel is 50 μm. (K) Sperm counts of wild-type and Carf –/– mice.Data are presented as the mean ± SEM, n = 6, ***P<0.001.

*Carf* mRNA and protein expression first presented during testis development (PD8 or earlier) and then increased at 12 days postpartum ( Figure 2A,B). To better understand the role of *Carf*, we created *Carf-*knockout mice via the CRISPR-Cas9 technique. Four small guide RNAs (sgRNAs) were created to construct *Carf-*knockout mice ( Figure 2C). The heterozygotes were generated by crossing the founders, and the offspring of wild-type, heterozygous type and *Carf-*knockout type mice were obtained after mating to heterozygous mice ( Figure 2D). Both the gene and protein levels demonstrated successful *Carf*/CARF knockout ( Figure 2E,F). We found that *Carf-*knockout male mice developed normally, but their fertility was severely impaired, mainly due to abnormal sperm development ( Figure 2G). In addition, we found that the size of the testes of *Carf*-depleted mice was smaller than that of their wild-type counterparts at 20 weeks of age but not at 8 weeks of age ( Figure 2H). The *Carf-*knockout males presented significantly reduced testis weight ( Figure 2I, *P* < 0.001, *n* = 6). We found that there were still a small number of spermatogonium and primary spermatocytes in some seminiferous tubules of *Carf* ^*–*/ *–*^ male mice, but in most seminiferous tubules, germ cells were completely absent, which resembled characteristic severe azoospermia ( Figure 2J). Moreover, unlike the wide-type mice, no sperm were identified in the epididymides of 20-week-old *Carf* ^*–*/ *–*^ mice ( Figure 2J,K). These data indicated that *Carf* is indispensable for the development of germ cells and spermatogenesis.

### CARF binds to the splicing factor PABPC1 and participates in alternative splicing

**Figure 3:**
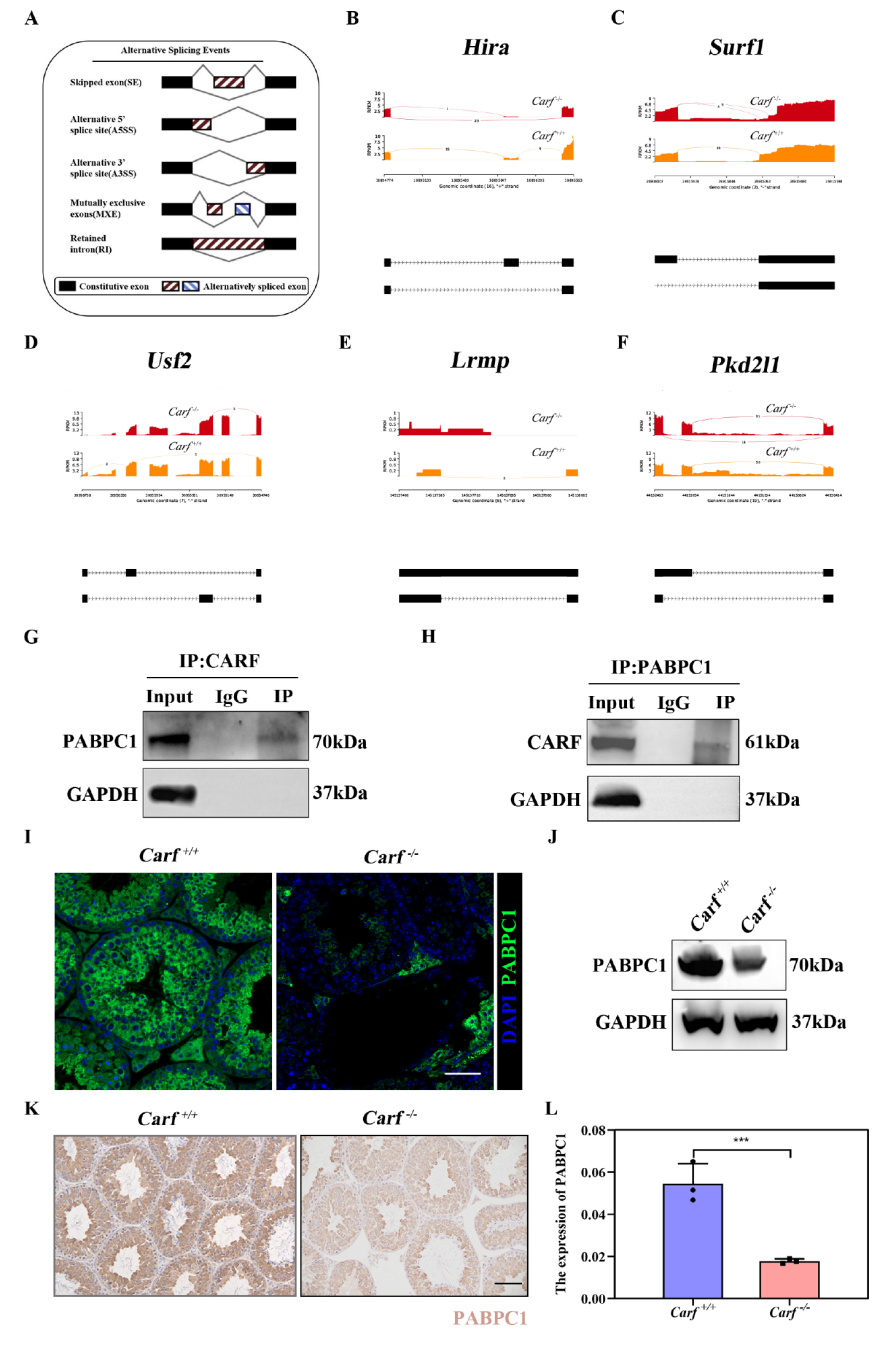
Figure 3 CARF interacts with PABPC1 to participate in RNA alternative splicing (A) Abnormal alternative splicing patterns, including skipped exons, alternative 5′ splice site (A5SS), alternative 3′ splice site (A3SS), mutually exclusive exons (MXE) and retained intron (RI) caused by Carf defects. (B) Abnormal variable splicing of the functional gene Hira, which is related to germ cell development caused by Carf defects. It belongs to the alternative 5′ splice site (A5SS) exception mode. (C) Abnormal variable splicing of the functional gene Surf1, which is related to germ cell development caused by Carf defects. It is an alternative 3′ splice site (A3SS). (D) Abnormal variable splicing of the functional gene Usf2, which is related to germ cell development caused by Carf defects. It belongs to the mutually exclusive exons (MXE). (E) Abnormal variable splicing of the functional gene Lrmp, which is related to germ cell development caused by Carf defects. It belongs to the retained intron (RI) family. (F) Abnormal variable splicing of the functional gene Pkd2l1, which is related to germ cell development caused by Carf defects. It belongs to the retained intron (RI) family. (G) Co-IP analysis of the interaction between CARF and PABPC1. PABPC1 expression was detected in the IP products of CARF, and IgG was used as a control. GAPDH served as a loading control. (H) Co-IP analysis of the interaction between CARF and PABPC1. CARF expression was detected in the IP products of PABPC1, and IgG was used as a control. GAPDH served as a loading control. (I) Immunostaining of PABPC1 in wild-type and Carf–/– testis sections (PABPC1: green); DAPI was used to stain the dye the nuclei, scale bar: 50 μm. (J) Western blot analysis of PABPC1 protein levels in testes from wild-type and Carf–/– mice. GAPDH served as a loading control. (K) Immunohistochemical analysis of the expression of PABPC1 in testes from wild-type and Carf–/– testis sections. Scale bar: 50 μm. (L) Quantitative results of (K). n = 3, Data are presented as the mean ± SEM. ***P < 0.001.

The conserved domains of CARF, including XTBD and DSRM, can serve as scaffolding modules and regulate the interactions between *Carf* and other proteins. To explore the role of *Carf* in the process of variable splicing, we conducted RNA sequencing (RNA-Seq) on testicular tissues from three wild-type mice and three *Carf*-knockout mice. The results revealed various forms of abnormal alternative splicing in *Carf* ^*–*/ *–*^ mice, such as skipping, alternative 5′ splice site (A5SS) and 3′ splice site (A3SS), mutually exclusive exons (MXE) and retained introns (RIs) ( Figure 3A). There are 23 junction reads of the Hira exon skipping isoform in *Carf* ^–/–^ and 1 junction read of the exon inclusion isoform. However, there are no junction reads of the exon skipping isoform in *Carf* ^+/+^. These results suggest that Hira exhibits SE aberrant alternative splicing in the testes of *Carf* ^–/–^ mice ( Figure 3B), which plays a key role in histone replacement of protamine [28]. In *Carf* ^–/–^, there are 3 junction reads for the exon skipping isoform and 9 junction reads for the exon inclusion isoform in *Surf*. In *Carf* ^+/+^, there are 16 junction reads for the exon skipping isoform and no junction reads for the exon inclusion isoform, indicating that *Surf1* presented abnormal A3SS variable splitting in *Carf* ^–/–^ mice ( Figure 3C). Mitochondrial proliferation is decreased in *Surf*-deficient mice, which affects spermatogenesis [29]. *Usf2* has abnormal variable splitting in the MXE form in *Carf* ^–/–^ mice. Five junction reads appeared in the second exon of *Carf* ^–/–^, and 3 junction reads appeared in the first exon of *Carf* ^+/+^ ( Figure 3D). *Usf2* regulates the process of spermatogenesis by maintaining normal support for cell differentiation [30]. *Lrmp* represents abnormal variable splicing of the RI form in *Carf* ^*–*/ *–*^ mice. There are 2 junction reads that support RI-type variable clipping in *Carf* ^–/–^ mice, whereas there are no junction reads that support RI-type variable clipping in *Carf* ^+/+^ mice ( Figure 3E). Lrmp promotes zygotic development by inducing centrosome nuclear attachment [31]. Pkd2l1, which regulates G protein signaling involved in the acrosome reaction and sperm production, is associated with abnormal variable splicing of the SE form in *Carf* ^–/–^ mice [32]. The *Pkd2l1* gene in *Carf* ^–/–^ mice has 14 junction reads for the exon skipping isoform and 31 junction reads for the exon inclusion isoform, whereas there are no junction reads for the exon skipping isoform in *Carf* ^+/+^ mice and 30 junction reads for the exon inclusion isoform ( Figure 3F). The abnormal alternative splicing of these genes is involved in germ cell development.

We used mouse testicular tissue for immunoprecipitation‒mass spectrometry analysis to identify the binding protein of CARF in wild-type and *Carf* ^–/–^ mice, and the results revealed a significant decrease in the binding of PABPC1 in *Carf* ^–/–^ mice ( Supplementary Figure S2C). The results of coimmunoprecipitation further confirmed the interaction between CARF and PABPC1 ( Figure 3G,H). We then compared the localization of PABPC1 in testicular tissue sections from wild-type and *Carf* ^*–*/ *–*^ mice and found that PABPC1 expression was largely decreased or even absent in *Carf* ^*–*/ *–*^ mice ( Figure 3I). This observation was further confirmed by western blot analysis ( Figure 3J). Immunohistochemical analysis of PABPC1 was performed in testicular tissue slices from wild-type and *Carf-*knockout mice, and the results revealed that PABPC1 expression decreased ( Figure 3K,L). These results strongly suggest that *Carf* is involved in the regulation of spermatogenesis through PABPC1, that PABPC1 plays a key role in the regulation of variable splicing, and that the role of *Carf* in variable splicing may be mediated by PABPC1.

### CARF regulates the ratio of pachytene-piRNA through the PABPC1-PIWIL1 axis

**Figure 4:**
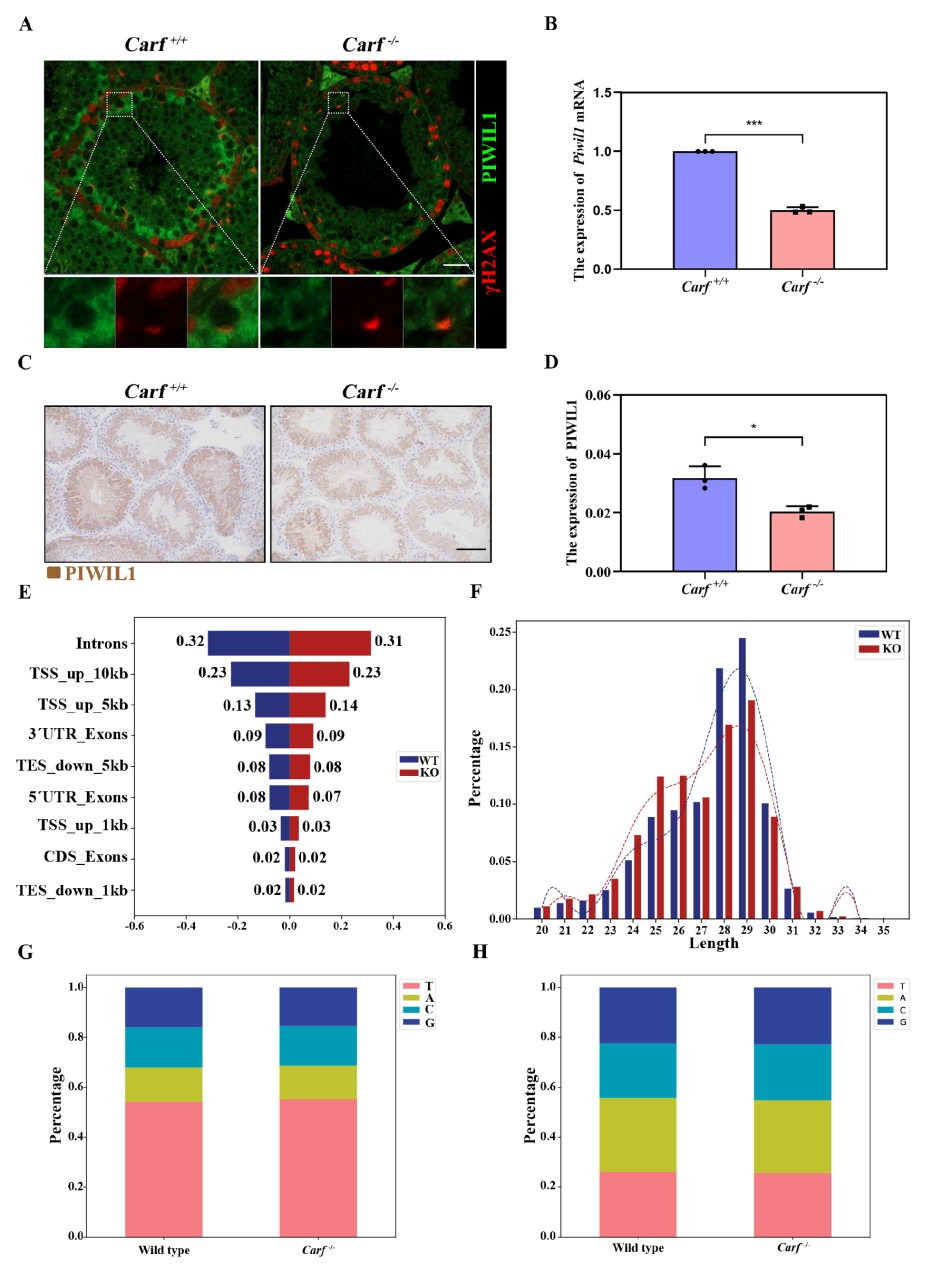
Figure 4 *Carf* defects inhibits the expression of PIWIL1 and piRNA maturation (A) Immunostaining of PIWIL1 in wild-type and Carf–/– testis sections (PIWIL1: green; γH2AX: red); γH2AX is used as a marker of DNA damage. Scale bar 50 μm. (B) PCR analysis of Piwil1 mRNA levels in testes from wild-type and Carf–/– mice. GAPDH served as a loading control. Data are presented as mean ± SEM. ***P < 0.001. (C) Immunohistochemical analysis of the expression of PIWIL1 in testes from wild-type and Carf–/– testis sections. Scale bar: 50 μm. (D) Quantitative results of (C). n = 3, Data are presented as the mean ± SEM. *P < 0.05. (E) Transcript and piRNA abundances in wild-type and Carf–/– testes are shown for illustrative examples from 16.5 dpp mice. (F) Analysis of the length distribution of piRNAs in wild-type and Carf–/– testes from P16.5 dpp mice. (G) The first nucleotides of the piRNAs, showing a strong U bias in wild-type and Carf–/– testes from P16.5 mice. (H) The tenth nucleotides of the piRNAs, showing a strong U bias in wild-type and Carf–/– testes from P16.5 mice.

Previous studies reported that PABPC1 directly binds to and participates in the regulation of PIWIL1 [ 17, 33 ]. Therefore, we further examined whether PIWIL1 expression is affected in *Carf* ^*–*/ *–*^ mice. Our experiments demonstrated that the expression of PIWIL1 in testicular tissue significantly decreased in *Carf* ^*–*/ *–*^ mice, and qPCR analysis indicated that *Piwil1* mRNA expression was also significantly decreased ( Figure 4A,B). Immunohistochemical analysis of PIWIL1 was performed in testicular tissue slices from wild-type and *Carf-*knockout mice, and the results revealed that PIWIL1 expression decreased ( Figure 4C,D). PIWIL1 is a major functional protein in the piRNA pathway. Next, we performed size and abundance profile analysis of small RNAs (15~50 nt) at 16 days postpartum (the meiotic pachytene stage) and sequenced them with libraries constructed from total RNA.

piRNA reads were mapped to the mouse genome, and the percentages of matched reads were similar between both groups, indicating good quality of the datasets ( Figure 4E). The data demonstrated that the ratio of pachytene-piRNA (27-32 nt) was lower in *Carf* ^*–*/ *–*^ testes than in control testes ( Figure 4F). The composition of the first nucleotide of piRNAs in *Carf* ^*–*/ *–*^ mice was then explored. Our results revealed that piRNAs from 24–32 nt reads of *the Carf* ^*–*/ *–*^ library presented a strong U bias similar to that of the control group ( Figure 4G), indicating that they were managed at the 5′ ends as mature piRNAs. Furthermore, 3′ end trimming, which was determined by comparing the perfect matches (i.e., precisely aligned 5’ ends), revealed that *Carf* deletion did not affect the 3′ terminal processing of piRNAs ( Figure 4H). These results indicated that *Carf* might affect the ratio of pachytene-piRNA.

## Discussion

Compared with other organs, alternative splicing occurs more frequently in the testis, especially during the development phase [34]. Multiple previous studies have shown that male infertility can be resulted from alternative splicing [35]. As regulators, RNA-binding proteins are critical in various splicing processes [36]. CARF is known to be involved in a variety of biological processes [37]. It was first discovered as a novel ARF-binding protein and participates in DNA damage and cell cycle checkpoint responses by regulating the ATM/CHK1/CHK2, p53 and ERK pathways [ 38, 39]. Previous studies also indicated that CARF could mediate spermatogonial self-renewal and proliferation through the Wnt pathway [27]. Interestingly, reactivation of the Wnt signaling pathway does not fully restore the fertility of *Carf* ^*–*/ *–*^ mice. Therefore, we believe that other important mechanisms may cause male sterility and germ cell loss in *Carf* ^–/–^ mice. Our data revealed that spermatocytes and RSs presented increased *Carf* expression compared with SG, ESs, and spermatozoa, indicating that *Carf* may be critical for maintaining polyadenylated mRNA storage and activation in these cells.

Our data demonstrated that the number of germ cells was reduced in *Carf* ^–/–^ mice at 20 weeks of age. Furthermore, we showed that CARF interacted with the splicing factor PABPC1 to participate in alternative splicing and indirectly regulated PIWIL1 expression as well as the ratio of pachytene-piRNA. However, we had limitations in terms of cell collection and processing, and we were not able to obtain sufficient numbers or purities of cells for RNA-seq analysis, which may have affected the accuracy of the results to some extent. However, our results suggest that abnormal alternative splicing could result in the loss of germ cells in *Carf* ^–/–^ mice. In fact, multiple genes that were isolated from *Carf* ^–/–^ mouse testes and related to spermatogenesis presented abnormal splicing patterns. Alternative splicing of genes such as *SPAG11B* [40], *SPAG11* [41] and *SPAG6* [42] plays important roles in spermatogenesis [43]. WT1 is an important regulator of normal spermatogenesis and can be modified with zinc finger 1 and replaced by +KTS to bind to transcription factors and splicing factors [44]. Cesari *et al*. [45] proposed that meiosis is controlled by a combination of Spo11 alternative splicing and splicing factor recruitment. RNA-binding protein 9 is also known to be associated with alternative splicing, which is critical for spermatogenesis. Furthermore, hnRNPH1 can recruit PTBP2 and SRSF3 to regulate alternative splicing in germ cells [46]. Overall, we propose that the main mechanism of germ cell loss we observed in this study might be the deletion of functional genes that directly affect gene splicing, which leads to the occurrence of abnormal selective splicing.

Alternative splicing can be caused by many factors, including abnormal expression of splicing factors, and often results in improper splice site recognition [47]. As shown in this study, we identified hundreds of unique alternative splicing patterns in *Carf* ^–/–^ testes, suggesting the regulatory role of CARF in this process. Further IP‒MS and Co-IP experiments verified our findings, which revealed that CARF interacts with the key splicing factor poly(A)-binding protein PABPC1. The poly(A)-binding protein PABPC1 plays a key role in translation regulation during spermatogenesis in a time-dependent manner [17]. Conserved PABPs bind specifically to poly(A) tails at the 3′ ends of mRNAs to regulate their translational activity in germ cells [48]. PABPC1 was first identified by Kimura M *et al*. [33] via mass–spectral analysis, which revealed that PABPC1 could facilitate the final maturation of sperm by regulating the post-transcriptional expression of an alternative exome [ 11, 49]. In addition, Oztuk *et al*. [50] reported that the expression of PABPC1 was decreased in testicular biopsy tissues from non-obstructive azoospermia patients, suggesting the critical role of PABPC1 in spermatogenesis. In this study, we found that the expression pattern of PABPC1 was similar to that of CARF, both of which are expressed in spermatocytes and are highly expressed in round spermatids (RSs). Recent studies have demonstrated that PABPC1 can participate in pre-mRNA processing in the nucleus as well as in mRNA metabolism in the cytoplasm [51]. Therefore, our results indicated that CARF could interact with PABPC1 and regulate pre-mRNA splicing. In addition, they play important roles in mRNA translational regulation at later stages of spermatogenesis.

Sawazaki R *et al*. [52]reported that PABPC1 can non-specifically bind to the mRNA poly (A) tail and directly bind to PIWIL1 (formerly known as MIWI) as well as other translation regulators. PIWIL1 binds to the 3′-untranslated regions (3′-UTRs) of several spermiogenic mRNAs via its N-terminal domain. The binding of PIWIL1 and PABPC1 is mediated through its N- and C-terminal domains in an RNA-dependent manner [17]. Therefore, this binding can be easily affected by other RNA-binding proteins.

A previous study revealed that decreased *Pabpc1* mRNA level is associated with the downregulation of overall piRNA-metabolic gene expression at both the transcriptional and post-transcriptional levels [53]. Given that CARF can interact with PABPC1, it is not surprising that the expression of PIWIL1 is also decreased in *Carf* ^–/–^ mice, along with an impaired ratio of pachytene-piRNAs (mature piRNAs). PIWIL1 is a key protein in the piRNA pathway and is required for robust accumulation of pachytene piRNAs [54]. Unlike other types of piRNA proteins, PIWIL1 can act as a small RNA-guided RNase (slicer) and requires extensive complementary pairing for target cleavage, at least *in vitro*. Disruption of its catalytic activity in mice with a single point mutation can lead to germ cell apoptosis and male infertility [55]. Importantly, no direct binding or interaction between CARF and PIWIL1 was identified in this study. Our results suggest that PABPC1-dependent CARF regulation is indispensable for maintaining normal spermatogenesis.

In summary, this study explored the role of *Carf* in the transcriptional regulation of alternative splicing during spermatogenesis. Our findings not only help to better understand the spermatogenesis process but also shed light upon the development of new therapeutic treatments for male infertility.

## Supporting information

supplementary_Figures

Supplementary_Table_2

Supplementary_Table_1

## Supplementary Data

Supplementary data is available at *Acta Biochimica et Biophysica Sinica* online.
