# Supplementary material for: CARF regulates the alternative splicing and piwi/piRNA complexes during mouse spermatogenesis through PABPC1: CARF regulates spermatogenesis through PABPC1
Source: Acta Biochim Biophys Sin (Shanghai). 2024 Dec 11;57(4):656–66. doi: 10.3724/abbs.2024224 (PMC12040762; doi:10.3724/abbs.2024224)
Supplement: supplementary_Figures [file supplementary_Figures.docx]

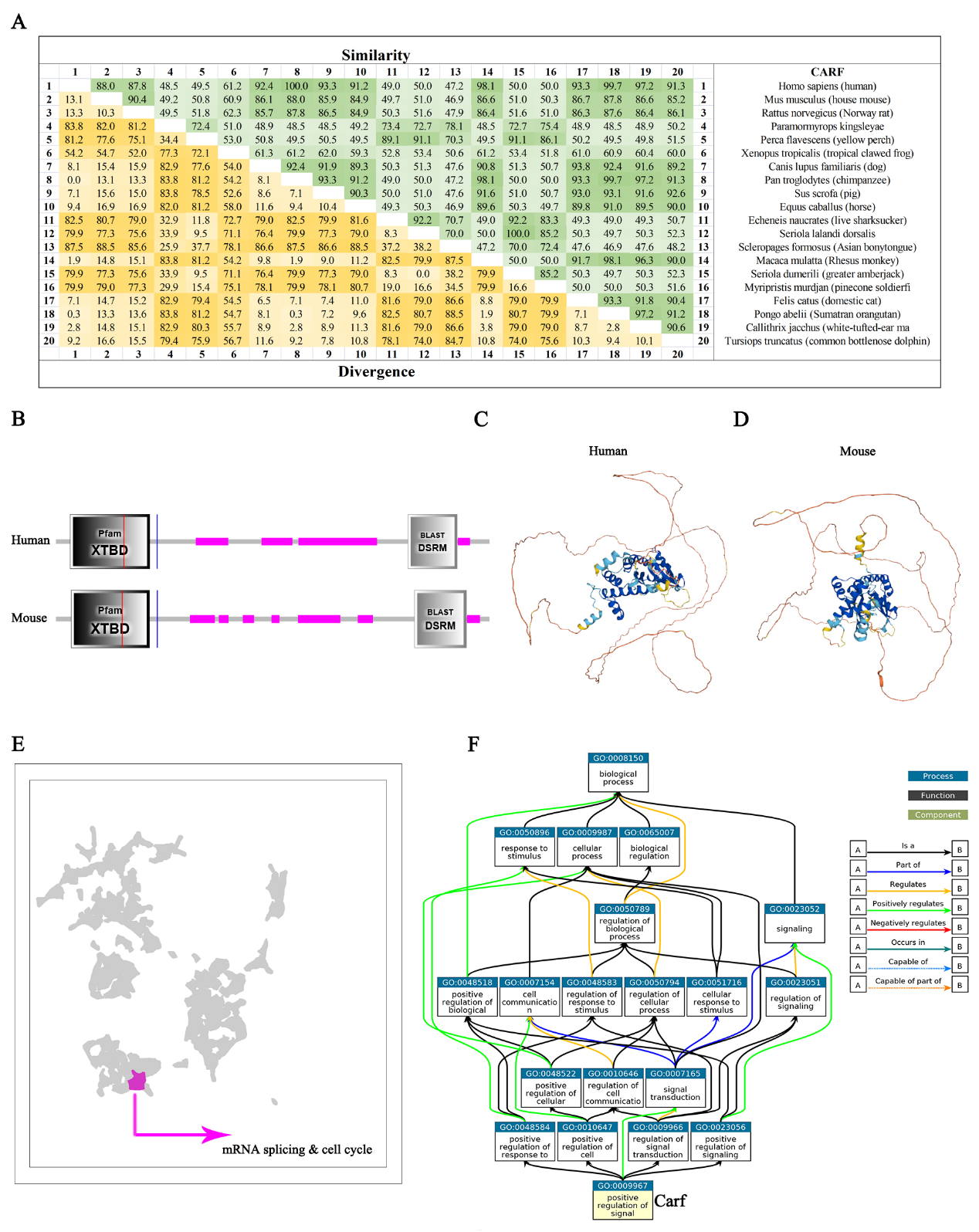


**Supplementary Figure S1. CARF is highly conserved and involved in transcriptional regulation** (A) Conservation analysis of the CARF protein sequence in humans and mice. The percentage obtained at the diagonal intersection position is the homology value of CARF between the two species. (B) Domain analysis of the CARF protein in humans and mice via online SMART software (<https://smart.embl-heidelberg.de/>). (C) Predictive structural analysis of the CARF protein in humans via AlphaFold software (https://alphafold.com/). (D) Predictive structural analysis of the CARF protein in mice via AlphaFold software (<https://alphafold.com/>). (E) Functional analysis of the human CARF protein (<https://www.uniprot.org/uniprotkb>). (F) Functional analysis of the mouse CARF protein (<https://www.ebi.ac.uk/QuickGO>).


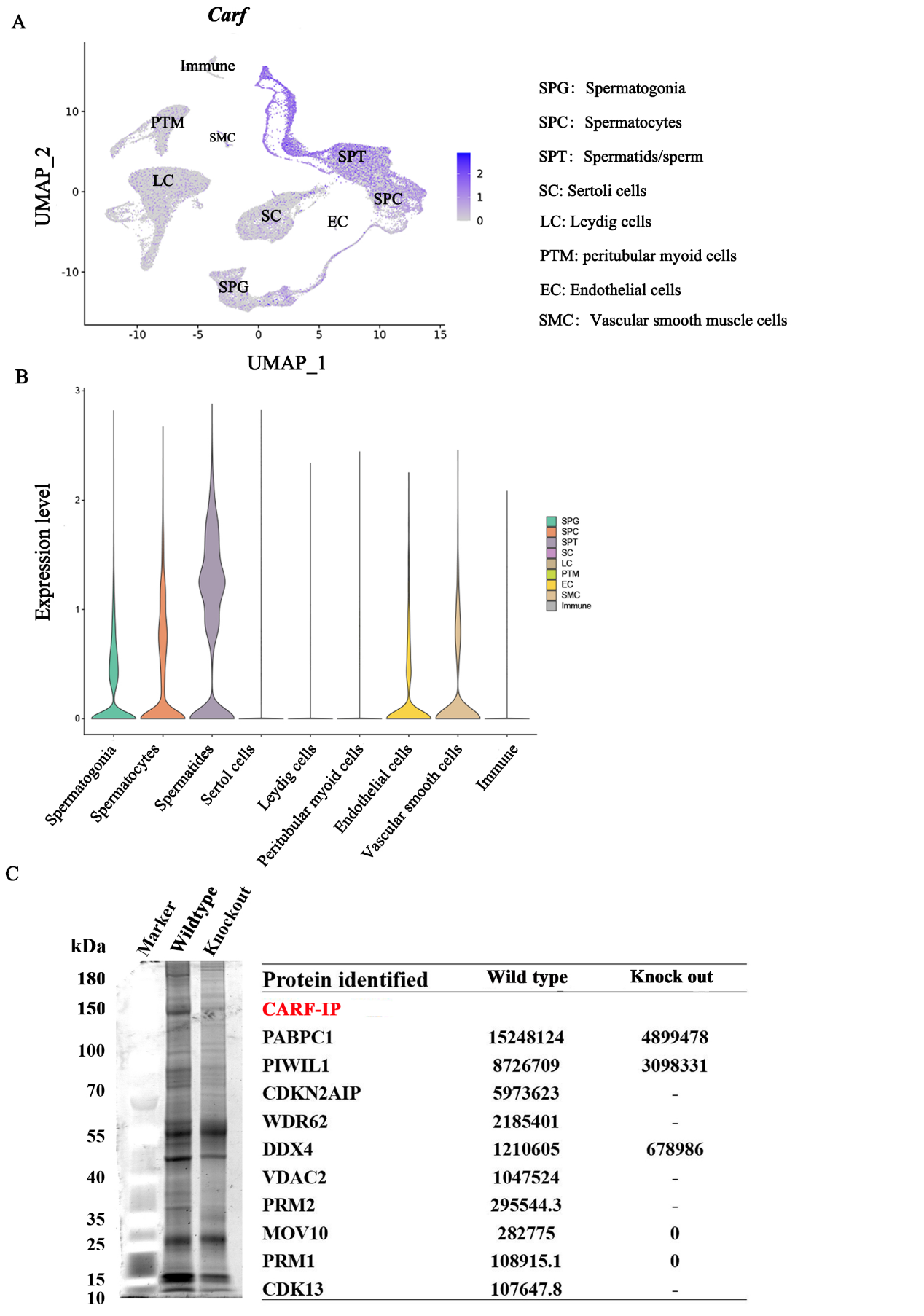


**Supplementary Figure S2. Expression profile of *Carf* in different types of germ cells and analysis of immunoprecipitation protein mass spectrometry results**  (A) Expression profile of *Carf* in different types of germ cells (<http://malehealthatlas.cn/>). (B) Expression levels of *Carf* in different types of germ cells (<http://malehealthatlas.cn/>). (C) A representative gel image showing bands representing proteins immunoprecipitated by the monoclonal anti-CARF antibody in testicular tissue from wild-type and *Carf-*knockout mice.
