# Supplementary material for: CARF regulates the alternative splicing and piwi/piRNA complexes during mouse spermatogenesis through PABPC1: CARF regulates spermatogenesis through PABPC1
Source: Acta Biochim Biophys Sin (Shanghai). 2024 Dec 11;57(4):656–66. doi: 10.3724/abbs.2024224 (PMC12040762; doi:10.3724/abbs.2024224)
Supplement: Supplementary_Table_2 [file Supplementary_Table_2.docx]

| **Supplementary Table S2. Antibodies used in this study** | | | | |
| --- | --- | --- | --- | --- |
| Antibodies | Species | Concentration | Cat # |  |
| CARF | Rabbit | 1:100(IF)1:1000(WB) | 16615-1-AP |  |
| PIWIL1/MIWI | Rabbit | 1:100(IF)1:1000(WB) | # 6915S |  |
| PABPC1 | Rabbit | 1:100(IF)1:1000(WB) | # 5940S |  |
| GAPDH | Mouse | 1:10000 | AC002 |  |
| γH2AX | Mouse | 1:200 | WH0003014M5 |  |
| Goat anti-mouse IgG | Goat | 1:10000 | AC011 |  |
| HRP Goat anti-mouse IgG | Goat | 1:10000 | AS066 |  |
| HRP Goat anti-rabbit-IgG | Goat | 1:10000 | AS014 |  |
| Goat Anti-Rabbit IgG H&L (Alexa Fluor® 488) | Goat | 1:400 | ab150077 |  |
| Goat Anti-Rabbit IgG H&L (Alexa Fluor® 594) | Goat | 1:400 | ab150080 |  |
