# Supplementary material for: CARF regulates the alternative splicing and piwi/piRNA complexes during mouse spermatogenesis through PABPC1: CARF regulates spermatogenesis through PABPC1
Source: Acta Biochim Biophys Sin (Shanghai). 2024 Dec 11;57(4):656–66. doi: 10.3724/abbs.2024224 (PMC12040762; doi:10.3724/abbs.2024224)
Supplement: Supplementary_Table_1 [file Supplementary_Table_1.docx]

| **Supplementary Table S1. Sequences of primers used in this study** | | |
| --- | --- | --- |
| Target | Sequence (5′ to 3′) | Application |
| gRNA target sequence | GTCAGCGCGGCCTGCACCGGCGG | gRNA target sequence |
|  | GAGGAGCCTTAGTTATGACCAGG |  |
|  | GAGGAGCCTTAGTTATGACCAGG |  |
|  | AGTTTATATCTAGAACCGGGTGG |  |
| *Carf* | F1: CTTTTGTTTATTTTGGCCGCGTG-3 | Genotyping |
|  | R1: TAGAAAGCCAGAAGGCCAAAAGTC |  |
|  | R2: CCCTAGGAAGACATGGTTCGC |  |
| *Pabpc1* | F: AGGGGATATGCTCCCCAACT | qRT-PCR |
|  | R: ACTGAATCCGGTGTTGCCAT |  |
| *Piwil1* | F: CTCCTGAAGCTGACCAAGGA | qRT-PCR |
|  | R: CTTGGTGAGGTTCAGGGGAT |  |
